# Type A Trichothecene Metabolic Profile Differentiation, Mechanisms, Biosynthetic Pathways, and Evolution in *Fusarium* Species—A Mini Review

**DOI:** 10.3390/toxins15070446

**Published:** 2023-07-05

**Authors:** Jianhua Wang, Mengyuan Zhang, Junhua Yang, Xianli Yang, Jiahui Zhang, Zhihui Zhao

**Affiliations:** 1 Institute for Agro-Food Standards and Testing Technology, Ministry of Agriculture, Shanghai Academy of Agricultural Sciences, 1000 Jinqi Road, Shanghai 201403, China; m210300781@st.shou.edu.cn (M.Z.); yangjunhua@saas.sh.cn (J.Y.); yangxianli@saas.sh.cn (X.Y.); jiahuizhang1224@163.com (J.Z.); 18918162068@163.com (Z.Z.); 2 College of Food Sciences & Technology, Shanghai Ocean University, Shanghai 201306, China

**Keywords:** mycotoxin, DAS, NEO, T-2, trichothecenes, *Fusarium* species

## Abstract

Trichothecenes are the most common *Fusarium* toxins detected in grains and related products. Type A trichothecenes are among the mycotoxins of greatest concern to food and feed safety due to their high toxicity. Recently, two different trichothecene genotypes within *Fusarium* species were reported. The available information showed that *Tri1* and *Tri16* genes are the key determinants of the trichothecene profiles of T-2 and DAS genotypes. In this review, polymorphisms in the *Tri1* and *Tri16* genes in the two genotypes were investigated. Meanwhile, the functions of genes involved in DAS and NEO biosynthesis are discussed. The possible biosynthetic pathways of DAS and NEO are proposed in this review, which will facilitate the understanding of the synthesis process of trichothecenes in *Fusarium* strains and may also inspire researchers to design and conduct further research. Together, the review provides insight into trichothecene profile differentiation and *Tri* gene evolutionary processes responsible for the structural diversification of trichothecene produced by *Fusarium*.

## 1. Introduction

Filamentous fungi within the *Fusarium* genus are the most important etiological agents of a variety of plant diseases worldwide, resulting in huge economic losses annually. Additionally, mycotoxins produced by these pathogens are also of concern due to their wide range of toxicological effects [1,2,3,4]. Among these toxic secondary metabolites, trichothecenes are the most commonly detected *Fusarium* mycotoxins, with relatively high contents compared with other ones [1,2,3,4]. Trichothecene is a large family of non-volatile sesquiterpenes that are classified into four different groups (type A, B, C, and D) according to structural variations [5,6]. To date, more than 200 trichothecenes have been identified in nature, which represent a major threat to food and feed safety [7].

**Figure 1:**
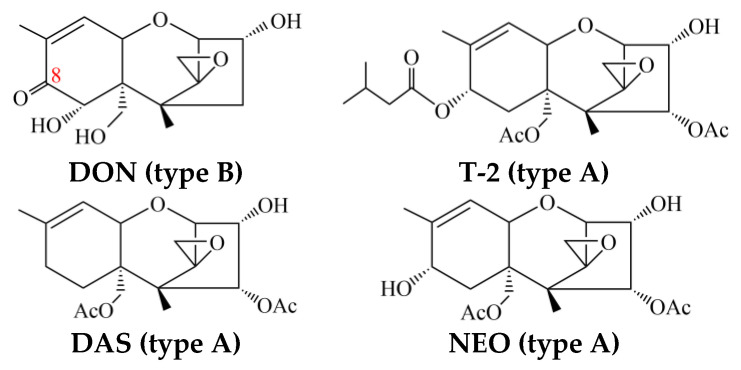
Chemical structures of Type A and B trichothecenes. Examples of type A trichothecenes include T-2 toxin, DAS, and NEO. DON is an example of a type B trichothecene.

*Fusarium* trichothecenes are divided into two major types characterized by the absence (type A trichothecenes) or presence (type B trichothecenes) of a keto group at carbon atom 8 (C-8). Type A trichothecenes contain an ester function (e.g., T-2 toxin), a hydroxyl group (e.g., neosolaniol, NEO) at C-8 of the skeleton 12,13-epoxytrichothec-9-ene (EPT) molecule, or no substituent at all at C-8 (e.g., 4,15-diacetoxyscirpenol, DAS) [5,6,7] (Figure 1). As illustrated in Figure 1, type B trichothecenes, such as deoxynivalenol (DON) and its acetylated derivatives, possess a keto group at C-8 [5]. Trichothecenes have been shown to be potent inhibitors of nucleotides and protein synthesis and can affect mitochondrial function, induce immunosuppression, etc., in eukaryotic organisms [8,9]. Trichothecenes can also act as virulence factors in plants, which will facilitate the colonization and spreading of the pathogens in host tissues [10,11,12]. The *Fusarium* trichothecenes of greatest concern are type A trichothecenes, with their high toxicity. 

This review seeks to outline recent findings on type-A-trichothecene-producing *Fusarium* species, the *Tri* gene’s genetic diversity, and evolution. In addition, current gaps, advances, and potential topics for future studies on *Fusarium* and trichothecenes are also mentioned. 

## 2. Metabolic Profile Differentiation in Type-A-Trichothecene-Producing *Fusarium*

It is well known that comprehensive research on the differentiation of type B trichothecenes has been conducted in the *Fusarium graminearum* species complex (FGSC), with the most important *Fusarium* species causing fusarium head blight in wheat around the world. In the FGSC, three different trichothecene genotypes (chemotypes) were distinguished according to their production of NIV (nivalenol), 3ADON (3-acetyldeoxynivalenol), or 15ADON (15-acetyldeoxynivalenol) [13,14]. FGSC species and trichothecene genotype diversity have been biogeographically structured worldwide. The statement suggests that the diversity of *Fusarium* species and trichothecene genotypes is not randomly distributed but instead follows geographic patterns [15,16,17]. This can have important implications for understanding the evolution and ecology of these organisms as well as for developing strategies for managing plant diseases caused by *Fusarium* species. Additionally, understanding the differences between different types of these mycotoxins is important for assessing their potential impact on human and animal health as well as for developing effective control and prevention strategies for reducing their occurrence in food and other agricultural products. 

Several *Fusarium* species, such as *Fusarium sporotrichioides*, *Fusarium poae*, *Fusarium kyushuense*, and *Fusarium langsethiae*, are well-known type A trichothecene producers. Among these fungi, different mycotoxin productivities were also observed. For example, *F*. *sporotrichioides* was reported to consistently produce T-2, HT-2, DAS, and NEO and has been recognized as the main source for T-2 and HT-2 [7,18,19]. The production of mycotoxins by 109 strains of *F*. *langsethiae* (23 strains), *F*. *poae* (49 strains), *F*. *sporotrichioides* (35 strains), and *F*. *kyushuense* (2 strains) was investigated independently in different laboratories [20]. From the compiled results, it was found that *F*. *langsethiae* and *F*. *sporotrichioides* consistently produced type A trichothecene (T-2, HT-2, and NEO). However, a different profile was observed in the 49 *F*. *poae* strains, and 41 of them produced type B trichothecenes (NIV and 4ANIV) in addition to type A trichothecenes (DAS). For the two *F*. *kyushuense* strains, no type A trichothecenes were detected from either of the strains [20]. However, among the mycotoxins produced by *F. poae*, NIV, a type B trichothecene, was cited as one of the most common mycotoxins produced by this species in the studies by [21,22,23,24]. *Fusarium armeniacum* was also reported to produce type A trichothecenes, such as T-2, DAS, and NEO [25].

In the past decade, several novel *Fusarium* species that produce type A trichothecenes have been reported, such as *Fusarium sibiricum* [26], *Fusarium palustre* [27], and *Fusarium goolgardi* [28,29]. *F*. *sibiricum* was mainly recovered in Siberia and the Russian Far East and formally described by Yli-Mattila et al. [26] in 2011. *F*. *sibiricum* is phylogenetically more closely related to *F*. *sporotrichioides* but is morphologically more similar to *F. poae* and *F*. *langsethiae* [26]. Analysis of trichothecene production revealed that all the tested *F. sibiricum* isolates could produce type A trichothecene T-2 as well as DAS with mean concentrations of 17.4 ppm and 0.2 ppm, respectively [26]. *F. palustre* is a new *Fusarium* species associated with the dieback of *Spartina alterniflora* in Atlantic salt marshes [27]. Subsequently, Rocha et al. [29] proved that strains from *F. palustre* can produce type A trichothecenes, including DAS, NEO, and T-2 toxin.

Despite the discovery of various metabolic profiles in type-A-trichothecene-producing species, no particular genotypes were outlined. The identification of two distinct genotypes within type A trichothecene producers was found in *F. goolgardi* [29]. *F*. *goolgardi* is an emerging species identified by Laurence et al. [28] from *Xanthorrhoea glauca* in natural ecosystems in Australia. Chemical analysis revealed the production of type A trichothecenes in *F. goolgardi* cultures [29]. Among the eight *F. goolgardi* strains evaluated, four of them (RBG5411, 5417, 5419, and 5420) produced T-2 toxin, DAS, NEO, and 8-acetylneosolaniol (hereinafter referred to as T-2 genotype in this work), while the other four strains (RBG6914, 6915, 5421, and 5422) produced only DAS (hereinafter referred to as DAS genotype in this work) [29]. So, the study by Rocha et al. [29] indicated that there were at least two distinct trichothecene genotypes in *F. goolgardi* populations. It is worth noting that a novel group of type A trichothecenes (NX toxins) produced by FGSC was identified by Varga et al. [30] in 2015. In this review, we will not delve into details about FGSC. To our best knowledge, only a single genotype has been reported for *F. langsethiae*, *F. sibiricum*, and *F. sporotrichioides*, and strains from these species can produce T-2 and some other type A trichothecenes, such as DAS and NEO. 

According to the present data, in general, type-A-trichothecene-producing *Fusarium* species may be indigenous and possibly endemic to their origin at a low frequency. If these strains become more abundant or are spread and exchanged widely through transportation and trade, type A trichothecenes could become a common contaminant in cereals and related products. For example, a high prevalence has been found for *F. langsethiae* on oats; however, it is now spreading even to barley cultivated in Mediterranean environments [31]. So, it will be important to monitor whether *Fusarium* species, such as *F*. *goolgardi*, have a selective advantage in specific ecosystems. Nevertheless, it is worth noting that the occurrence of type-A-trichothecene-producing *Fusarium* species in different geographic locations in the world suggests their wide distribution in nature. Novel type-A-trichothecene-producing species may be identified in further studies with more extensive collections in the future.

## 3. *Tri1* and *Tri16* Genes Are the Key Determinants of Trichothecene Profiles

The biosynthetic pathway and molecular regulation mechanism of trichothecenes are now relatively clear, and many studies have been conducted since the 1990s. Up until now, 15 trichothecene biosynthesis genes (*Tri* genes) have been identified and characterized in the *Fusarium* genome (Table 1). Molecular genetics revealed that these *Tri* genes occur at three loci. The 12-gene core locus on chromosome 2 is located within a 25 kb region as a cluster response for the synthesis of the EPT skeleton molecule and subsequent modifications at C-3, C-4, and C-15. The *Tri1*–*Tri16* locus on chromosome 1 is essential for the hydroxylation and acylation of C-8, respectively. The single-gene locus on chromosome 4, *Tri101*, is responsible for acetylation of the hydroxyl group at C-3, converting isotrichodermol to isotrichodermin. This step has been proven to serve as a mechanism for the self-protection of the trichothecene-producing organism [32], which can significantly reduce the toxicity of trichothecenes.

In trichothecene-producing *Fusarium* species, the *Tri1*–*Tri16* locus determines type A versus type B trichothecene production. In T-2 producers, *Tri1* and *Tri16* are responsible for the specific hydroxylation and acylation, respectively, at the C-8 position [33,34,35]. However, in type B trichothecene producers, the enzyme encoded by the *Tri1* gene catalyzes the hydroxylation of trichothecenes at both C-7 and C-8, but the *Tri16* gene is non-functional due to the presence of frameshifts and stop codons in its coding region [36]. Meanwhile, a non-functional *Tri16* in type-A-trichothecene-producing *Fusarium* strains should prevent the acylation of the hydroxyl of trichothecene intermediates at the C-8 position, and a non-functional *Tri1* gene should equally prevent hydroxylation of the C-8 and, of course, prevent the later acylation reaction catalyzed by the Tri16 enzyme. 

Sequence analysis of the *Tri1*–*Tri16* locus in four *F. sibiricum*, seven *F. langsethiae*, and six *F. sporotrichioides* revealed that the orientation and order of the two genes were the same as previously characterized for *F. sporotrichioides*, although the length of the *Tri1*–*Tri16* intergenic region varied among and within species [26]. According to the phylogenetic analysis of the *Tri1* and *Tri16* gene coding sequences, the two genes in *F. sibiricum* strains are more closely related to those of *F. sporotrichioides*. In *F. sibiricum*, the *Tri1*–*Tri16* locus is more similar in organization and sequence to those of *F. langsethiae* and *F. sporotrichioides* than to that in the species of *F. poae* [26]. 

**Figure 2:**
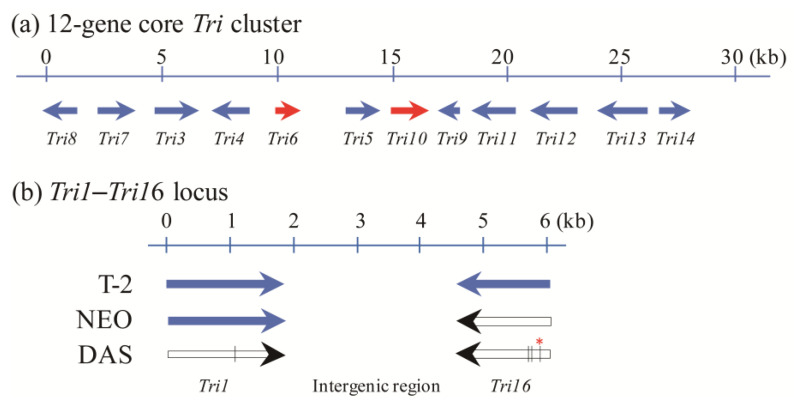
Type A trichothecene biosynthetic loci in *Fusarium* species. (**a**) The 12-gene core *Tri* cluster. (**b**) Comparison of the *Tri1*–*Tri16* locus in *Fusarium* species with T-2, NEO, and DAS genotypes, respectively. *Tri6* and *Tri10*, the two transcription factors, are shown in red. Arrows indicate genes and the direction of transcription. Filled arrows indicate that the *Tri* genes are functional, while the non-functional genes are indicated with empty arrows. Premature stop codons are indicated by vertical lines on the arrows; the frameshift that occurred in the *Tri16* gene in the DAS genotype is indicated above the panel by * and together with a vertical line on the arrow.

Recently, two different trichothecene metabolic profiles were identified in *F. goolgardi* strains. To reveal the reason why this phenomenon exists, the nucleotide sequences of different *Tri* genes from several type-A-trichothecene-producing *Fusarium* species were analyzed and compared with the two *F. goolgardi* groups. The results showed that no major differences were observed in the coding regions of the core cluster genes (including *Tri3*–*Tri8*, *Tri11*, *Tri13*, and *Tri101*) among these strains [29]. However, significant differences were identified in the *Tri1* and *Tri16* sequences. As shown in Figure 2, there is a transition (C-to-T) in the coding region of the *Tri1* gene, which resulted in a premature stop codon in the gene of the *F. goolgardi* strain with the DAS genotype [29]. According to previous studies, in T-2-producing *Fusarium* species, *Tri16* is an intronless gene [26]. In comparison with *F. sporotrichioides*, the *Tri16* coding region of DAS-genotype strains exhibited a single-nucleotide deletion, which introduced a frameshift mutation and caused two premature stop codons in the gene of the *F. goolgardi* strain with the DAS genotype. However, *Tri1* and *Tri16* orthologs from all the T-2 producers, including the *F. goolgardi* T-2 genotype strains, did not contain the same or any other similar nonsense or frameshift mutations in the coding regions [29]. Overall, the results showed that the *Tri1* gene is essential for the hydroxylation of type A trichothecene at C-8, and this gene determines the production of DAS, NEO, and T-2 toxins in *F. goolgardi* [29]. 

In the previous studies by Brown et al. [34], Peplow et al. [35], and Proctor et al. [36], the *Tri16* gene was found to be truncated in the *F. poae* strains examined, which would explain why some *F. poae* strains cannot produce T-2 toxin. Moreover, the contradictory reports about the ability of different geographically originated *F. poae* strains to produce type A trichothecenes may also be the cause of the misidentification of *Fusarium* species due to their high morphological similarity [18,19,26].

The organization and genomic context of the trichothecene biosynthetic locus *Tri1*–*Tri16* are similar in *F. langsethiae*, *F. sibiricum*, *F. sporotrichioides*, and *F. goolgardi* (including both the T-2 and DAS genotypes), but significantly different from those described for some of *F. poae* [26,29,36]. On the other hand, the occurrences of nonsense mutation and frameshift mutation in the coding region of *Tri1* and *Tri16* genes [29], respectively, led to the loss of functions of the two genes in type A trichothecene producers, such as *F. goolgardi* strains that possess a strict DAS genotype (Figure 2). These results indicate that we still have a lot to do about trichothecene biosynthesis in the *Fusarium* genus and also suggest the necessity of re-explaining the diversity of trichothecene production in these complicated fungi. Based on the research of the past, we can hypothesize that *Fusarium* strains that produce only trichothecene with a hydroxyl at C-8 may be identified in the future.

## 4. Proposed Biosynthetic Pathways of DAS and NEO and Comparisons with T-2

The biosynthesis of trichothecenes begins with the cyclization of the precursor substance trans-farnesyl pyrophosphate (FPP) to form trichodiene, followed by oxygenation, isomerization, cyclization, and esterification to finally form trichothecene toxins with various structures. The types and chemical structures of trichothecene toxins are mainly determined by the metabolic pathways involved and the genetic differences in *Tri* genes. According to the chemical structures of trichothecenes and even some newly identified trichothecene orthologs produced by *Fusarium* species, the biosynthetic pathways and gene functions can probably be predicted based on our existing understanding of trichothecene biosynthesis [37]. Comprehensive studies have been conducted to reveal the biosynthesis of T-2 since the 1990s, and its biosynthetic pathway and molecular regulation mechanism are now relatively clear. To our best knowledge, however, limited information is available for the other type A trichothecenes, such as DAS and NEO. 

Structurally, there are two hydrogen atoms at C-8 in the DAS molecule, while one of the two hydrogen atoms is replaced by a hydroxyl group and an isovalerate group, respectively, in NEO and T-2. That means the DAS genotype strains do not have the ability to synthesize the enzymes that can catalyze the hydroxylation and isovalerate addition to C-8. On the other hand, co-occurrence of DAS, NEO, and T-2 toxins in a single strain demonstrated that all the 3-acetylneosolaniol, 3,4,15-triacetoxyscirpenol, and 3-acetyl T-2 can be served as substrates of Tri8 (an esterase) by which the C-3 acetyl group is replaced by a hydroxyl [38]. The differential activity of Tri8, as defined by the DNA sequence, determines the production of either 3ADON or 15ADON in FGSC [39]. However, so far, it is still unclear whether strains with a type A trichothecene genotype that produces only NEO but no trichothecene with isovalerate function at C-8 naturally exist in the genus *Fusarium*. 

As shown in Table 1, the specific functions of most *Tri* genes in trichothecene biosynthesis have been studied in *Fusarium* species [33,35,40,41,42,43,44,45,46,47,48], which makes it possible to predict the biosynthetic pathway of different trichothecene metabolites, such as DAS and NEO. For example, the *Tri1* genes in T-2 producers are responsible for oxidation at C-8 of the trichothecene scaffold [33]. Target gene disruption of the *Tri1* gene blocks production of C-8-oxygenated trichothecenes and leads to the accumulation of DAS in *F. sporotrichioides* [33]. The recently identified DAS producer that carries a non-functional *Tri1* gene in *F. goolgardi* species further confirmed our thinking [29]. The data by Peplow et al. [35] indicate that *Tri16* encodes an acyltransferase that catalyzes the formation of ester side groups at C-8 during T-2 biosynthesis in *F. sporotrichioides*. Similarly, *Fusarium* strains with type A trichothecene genotypes containing a non-functional *Tri16* gene may produce NEO. 

**Figure 3:**
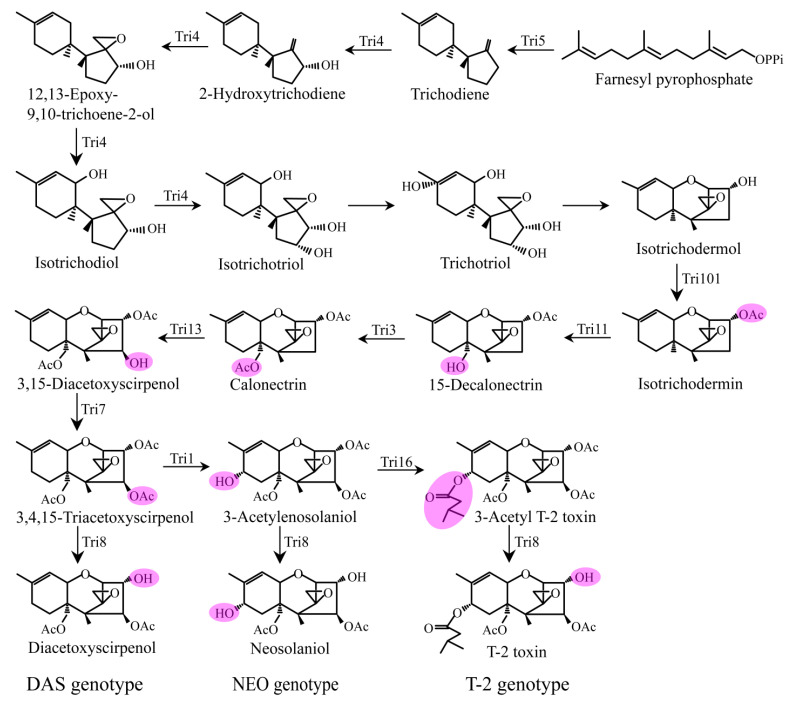
The proposed biosynthetic pathways of DAS, NEO, and their comparison with T-2 in *Fusarium*. Steps catalyzed by Tri enzymes are identified near the arrow showing the step. Unlabeled arrows indicate steps for which the specific genes or enzymes are unknown.

Based on the findings of trichothecene biosynthesis in *Fusarium*, as shown in Figure 3, we proposed the biosynthetic pathways of DAS and NEO and made a comparison with the T-2 biosynthetic pathway. As reviewed by Chen et al. [37] and Chen et al. [49], in trichothecene biosynthetic pathways, the reaction steps catalyzing FPP to calonectrin (CAL) are shared among *Fusarium* species. For detailed information on type A and type B trichothecene biosynthesis, please refer to previous publications [6,7,50,51,52]. In T-2 producers, intermediate metabolite CAL, which is eventually converted to T-2 toxin, undergoes a series of steps catalyzed by Tri13-Tri7-Tri1-Tri16-Tri8 sequentially. As we predicted, the same reactions occurred in the immediate two following steps, catalyzed by Tri13 and Tri7, respectively, after CAL during the biosynthesis of DAS, NEO, and T-2. CAL is hydroxylated by Tri13 at the C-4 position to produce the intermediate metabolite 3,15-diacetoxyscirpenol, and the hydroxyl group is subsequently converted to an acetyl group by the enzyme of Tri7 to produce 3,4,15-triacetoxyscirpenol (Figure 3). As mentioned above, the DAS strains have a pseudo-*Tri1* gene, so differences arise in the later steps after 3,4,15-triacetoxyscirpenol during the biosynthesis of DAS, NEO, and T-2. 

As shown in Figure 3, in DAS producers, 3,4,15-triacetoxyscirpenol is deacetylated by esterase encoded by *Tri8*, leading to the formation of DAS. In NEO and T-2 producers, 3,4,15-triacetoxyscirpenol is further converted to 3-acetylenosolaniol through the activity of the Tri1 enzyme. The product 3-acetylenosolaniol is deacetylated by the enzyme of Tri8 at C-3 to produce NEO. In light of this, we draw the conclusion that CAL is catalyzed via the Tri13-Tri7-Tri8 and Tri13-Tri7-Tri1-Tri8 pathways, respectively, during the biosynthesis of DAS and NEO. It is easy to understand that T-2 producers can also produce portions of DAS and NEO which are the intermediates of T-2 biosynthesis, since the T-2 strains contain all the functional *Tri* genes required for DAS and NEO biosynthesis. However, the definite biosynthetic pathway and detailed regulation mechanisms of DAS and NEO are unclear, and systematic studies still should be conducted, especially using the strict DAS genotype strains identified, such as the DAS-genotype *F. goolgardi* strains. Moreover, the proposed biosynthetic pathways of DAS and NEO in this work will provide new insights into trichothecene biosynthesis and guide researchers to carry out more extensive studies on this topic.

## 5. Evolution Potential of Type A Trichothecene Metabolic Profile Differentiation in *Fusarium*

Studies of trichothecene-producing *Fusarium* species indicate that the evolutionary process of the *Tri* loci is complex in fusaria and suggest that gain or loss functions, mutations, translocations, and non-functionalization occurred within and between *Tri* loci [23,36,39,53]. The structure diversity of trichothecenes is the cause of genetic polymorphism in the *Tri* genes. It was found that the evolution of *Tri* genes does not always correlate with the evolutionary process of *Fusarium* species, which has been maintained through balancing selection and accompanied by the evolution process of the fungi [54]. The studies by Proctor et al. [36] and Kelly et al. [55] reported inconsistencies between species phylogenies and *Tri1*–*Tri16*-based phylogenies. Specifically, trans-species evolution and genomic translocations of the *Tri1* gene have been identified, and this gene is found in at least four genomic contexts [36]. Recently, Kelly et al. [55] revealed that the evolution of a novel trichothecene-producing population in FGSC was accompanied by a marked change in selective pressure on *Tri1*. However, the genomic context and evolutionary affinities of the *Tri1* variants from type-A-trichothecene-producing strains have not been investigated. A wide range of sequencing and phylogenetic analyses of *Tri1* from diverse *Fusarium* strains is warranted to further reveal the origins and evolutionary processes of the type-A-trichothecene-producing strains with different genotypes. 

Proctor et al. [36] have also suggested that the *Tri1*–*Tri16* locus was the ancestral character state in the ancestral trichothecene-producing *Fusarium* species, and the gene was probably functional in the ancestral strains, as it is more likely for a gene to lose functionality than for a non-functional gene (such as due to deletions and nonsense mutations, etc.) to become functional [29,36]. So, we hypothesize that the two genotypes within *F. goolgardi* evolved from the same ancestor. The *Tri1* gene in *F. goolgardi* strains with the T-2 genotype is probably ancestral to the allele in strains with the DAS genotype. 

Nevertheless, it is worth noting whether strains that primarily produce NEO or co-occurrences of NEO and DAS without T-2 exist in nature or not. If this is the case, three genotypes will be classified within type A trichothecene strains. To simplify the description of type A trichothecene genotypes, we recommend using DAS, NEO, and T-2, respectively, for the strains, which will facilitate the implementation of scientific research and academic exchanges.

The results of previous studies provide evidence for a complex evolutionary process of *Tri* loci and specific *Tri* genes that included gain, loss, functional changes, rearrangement, and trans-species polymorphism [23,36,39,53]. The structural diversity of trichothecenes potentially reflects differences in selection pressure experienced by the fungi that produce the analogs [54]. Ward et al. [54] concluded that trichothecene structural diversity in the FGSC has been maintained through balancing selection. Thus, further investigations are required to reveal the important evolutionary event that has given rise to type A trichothecene structural differences through comparative analyses of different *Fusarium* species. These results will provide new insights into genetic basis changes or biochemical alterations that occurred in trichothecene biosynthesis and regulation as fungi with the pathways adapt to various environmental conditions. Multispecies comparisons of *Tri* loci and *Tri* genes may also provide key insights into the evolution process of trichothecene metabolism in *Fusarium*.

## 6. Conclusions and Future Prospects

Two major type-A-trichothecene-producing *Fusarium* groups were identified in nature: one group can produce trichothecene containing an ester function at the C-8 position and is represented by T-2; the other group produces trichothecene without a substituent at C-7 and C-8 but not T-2 and is represented by DAS. The phylogenetic relationship assessment of *Tri* genes provided important evidence for the genetic basis of chemotype differentiation within this species. The *Tri1*–*Tri16* locus is responsible for the chemical structure variation of these two genotypes; both *Tri1* and *Tri16* are functional in the T-2 genotype but non-functional in *Fusarium* strains with the DAS genotype due to the occurrence of premature stop codons caused by a point mutation within their coding regions [29]. The apparent genetic changes within type-A-trichothecene-producing *Fusarium* species highlight the need for monitoring and more phenotypic characterization of trichothecene-producing populations. 

As previously reviewed, the *Fusarium* genus and trichothecene genotype diversity vary significantly among different hosts and geographic locations [15,16,17]. Further investigations are required to track the spread of different trichothecene genotypes and to elucidate potential differences in their competitive abilities, including environmental adaptability and aggressiveness in different plant hosts. The environmental drivers of trichothecene metabolic profile differentiation in *Fusarium* are waiting to be further revealed, and continuous studies will be required to elucidate the ethology, host preference, economic loss caused, forecast and prediction, and control methods of different *Fusarium* populations. Most importantly, the molecular mechanisms of DAS and NEO biosynthesis should be comprehensively clarified. 

## Figures and Tables

**Table 1:** Function of *Tri* genes and major phenotype of individual *Tri* gene disruption in Type A trichothecene biosynthesis in *F. sporotrichioides*.

*Tri* Gene	Function	Mutant Phenotype
*Tri8*	C-3 deacetylase	3-acetyl T-2
*Tri7*	C-4 acetyltransferase	HT-2
*Tri3*	C-15 acetyltransferase	15-decalonectrin, 3,15-didecalonectrin
*Tri4*	multifunctional oxygenase	trichodiene
*Tri6*	zinc finger transcription factor	low levels of trichodiene
*Tri5*	trichodiene synthase	no trichothecenes
*Tri10*	regulatory gene	no trichothecenes
*Tri9*	unknown	not determined
*Tri11*	C-15 hydroxylase	isotrichodermin
*Tri12*	trichothecene efflux pump	no trichothecenes
*Tri13*	C-4 hydroxylase	8-hydroxycalonectrin, 4-deoxy T-2, 8-hydroxy-3-deacetylcalonectrin
*Tri14*	virulence factor	T-2
*Tri1*	C-8 hydroxylase	DAS
*Tri16*	C-8 acyltransferase	NEO, DAS
*Tri101*	C-3 acetyltransferase	isotrichodermol

## Data Availability

Not applicable.
